# Whey Protein *(Ma’aljobon)* as a Complementary Therapy for Treatment of Attention-deficit/ Hyperactivity Disorder (ADHD): A Randomized Open-label Controlled Clinical Trial

**DOI:** 10.31661/gmj.v9i0.1690

**Published:** 2020-08-27

**Authors:** Zeinab Mostajeran, Seyed Hamdollah Mosavat, Mostafa Najafi, Majid Emtiazy, Mohammad Hashem Hashempur

**Affiliations:** ^1^Department of Persian Medicine, School of Persian Medicine, Shahid Sadoughi University of Medical Sciences, Ardakan, Yazd, Iran; ^2^Pharmaceutical Sciences Research Center, Shiraz University of Medical Sciences, Shiraz, Iran; ^3^Research Center for Psychiatry and Behavior Science, Shiraz University of Medical Sciences, Shiraz, Iran; ^4^Behavioral Sciences Research Center, Al-Zahra Hospital, Isfahan University of Medical Sciences; ^5^The Research Center of Persian Medicine, Shahid Sadoughi University of Medical Sciences, Yazd, Iran; ^6^Noncommunicable Diseases Research Center, Fasa University of Medical Sciences, Fasa, Iran; ^7^Department of Persian Medicine, School of Medicine, Fasa University of Medical Sciences, Fasa, Iran

**Keywords:** Attention Deficit Disorder with Hyperactivity, Whey Proteins, Complementary Therapies, Integrative Medicine, Traditional Persian Medicine

## Abstract

**Background:**

Attention deficit hyperactivity disorder (ADHD) is a common, chronic, neurodevelopmental disorder that manifests in childhood with symptoms of hyperactivity, inattention, and impulsivity. *Ma’aljobon* (a kind of whey protein) that is derived from milk during cheese producing process is a popular dietary traditional product supposed to provide immune modulation and prevent neuropsychiatric disorder. We aimed to evaluate the efficacy of *ma’aljobon* in management of Attention-deficit/hyperactivity disorder (ADHD).

**Materials and Methods:**

In this open-label randomized, double arm, and controlled clinical trial, sixty four patients with ADHD who referred to out-patient child and adolescent clinic of *Khorshid* Hospital of *Isfahan*, Iran, were randomly assigned in the intervention group (*ma’aljobon* 25 g once daily plus standard conventional treatment (SCT)) or control group (SCT only) for a period of 8 weeks. Scores of the Strengths and Difficulties Questionnaire (SDQ) and Conners’ Continuous Performance Test (CPT) were set as the outcome measures.

**Results:**

Parent reported hyperactivity scale of SDQ showed a significant decrease in the intervention group compared to the control group (P=0.04). However, no significant between groups differences were observed in other scales of parent-reported SDQ. Also, according to the results of CPT, there was a significant improvement in the intervention group regarding attention and focus score (P=0.01).

**Conclusion:**

*Ma’aljobon* might be considered as a complementary remedy for improving hyperactivity, attention and focus of children with ADHD. However, further researches with larger sample size and longer duration should be done for achieving more reliable results.

## Introduction

Attention deficit hyperactivity disorder (ADHD) is a common, chronic, neurodevelopmental disorder that manifests in childhood with symptoms of hyperactivity, inattention, and impulsivity [1]. It affects 5% to 9% of school-aged children. ADHD adversely affects behavioral, cognitive, emotional, and social functions. Moreover, it can lead to secondary problems during adulthood including academic underachievement, increased risk of serious accidents, underemployment, and premature mortality [2]. Etiology of ADHD is complicated, not fully determined, and has a multi-factorial origin. Genetic, environmental, and neural factors might be involve in the neurobiologic readiness for ADHD [3]. Treatment is multimodal, often using different educational, behavioral and pharmacologic modalities [4-6]. Unfortunately, its medications have several side effects and need careful monitoring. Moreover, educational and behavioral modalities are expensive and time consuming. Therefore, the trend to use complementary and alternative medicine for management of ADHD is increasing [7-11]. *Ma’aljobon* is a kind of whey protein that is traditionally used in Persian medicine for different diseases [12]. Whey that is a protein complex derived from milk during cheese producing process is considered as a functional food with numerous health benefits. Today, whey protein is a popular dietary product supposed to provide immune modulation [13-15] and prevent neuropsychiatric disorder [16-19]. The lactoferrin, beta-lactoglobulin, alpha-lactalbumin, glycomacropeptide, and immunoglobulins are the main biological components of whey. It has immune-enhancing properties and is able to act as an antioxidant, antitumor, hypolipidemic, antihypertensive, antibacterial, antiviral, and chelating agent [17,20,21]. A previous study have showed that whey protein consumption decreased the cortisol concentrations, and prevented depressive feelings during acute stress through increasing serotonin [22]. Another study concluded that whey protein improved cognitive performance in stress-vulnerable subjects via increased brain tryptophan and serotonin activities [23]. However, the efficacy of this dairy remedy has not been previously evaluated in ADHD. Therefore, we designed a controlled, open-label, randomized clinical trial to evaluate the safety and efficacy of *ma’aljobon* in management of ADHD.

## Materials and Methods

###  Trial Design

In this open-label randomized, controlled clinical trial, we investigated the safety and efficacy of *ma’aljobon* powder in management of children with ADHD.

###  Participants

 This study was carried out in the outpatient child and adolescent clinic of Khorshid Hospital of Isfahan, Iran from October 2016 to May 2017. The inclusion criteria in this study were school children (both of boys and girls) aged 6 to 13 years with a confirmed clinical diagnosis of ADHD (according to Diagnostic and Statistical Manual of Mental Disorders -5 criteria). Informed consent form was signed by parents to enroll their child in the study. The exclusion criteria were having any significant physical impairment, history of a pervasive developmental disorder, schizophrenia, bipolar disorder, severe depressive episode, epilepsy or heart disease, and intelligence quotient (IQ) below 80.

###  Intervention

At first, the children were visited by a pediatrician and cases of ADHD were diagnosed. All eligible children with ADHD were divided into two parallel groups. Patients were randomly assigned to receive either *ma’aljobon* powder (as a complementary intervention) for two months (Nowak Pharmaceutical Co., Iran) (25 g in 100 cc water, once daily after breakfast) as the intervention group, or receive no intervention as the control group. Consumption of less than 70% of *ma’aljobon* powder during the trial was considered as noncompliance. It should be noted that children in both groups continued their previous standard conventional ADHD medications. Additionally, there was no co-intervention (e.g. nutritional recommendation).

###  Outcome Measures

The subscales scores of the Strengths and Difficulties Questionnaire (SDQ) and Conners’ Continuous Performance Test (CPT) were set as the primary outcome measures. SDQ is a multi-informant (parents, teachers, and older children) behavioral screening questionnaire for children and adolescents aged 2- 17 years old. It measures emotional symptoms, conduct problems, hyperactivity, peer problems, and prosocial behavior. It should be noted that SDQ was designed by Robert Goodman and has now been translated into several languages, including Persian. Reliability and validity of the Persian version of SDQ are reported in previous studies [24]. Additionally, CPT was used to analyze the problems of inattention. Scores of omission and commission indicate the participants’ performance in this test. Reliability and validity of the Persian version of CPT have been reported in previous studies [25].

###  Sample Size

According to previous similar studies [26,27] and by taking two-sided significance level of 0.05 and power of 80% into account, the sample size was calculated 29 participants in each group, and with a 10% drop rate, this number reached 32 participants in each group.

###  Safety Assessment

 Before selecting the sample by questioning the parents and reviewing the children’s medical records, we made sure that there was no sensitivity to dairy products. All of the parents were requested to inform the research team of any side effects, including gastrointestinal problems (such as bloating, diarrhea and constipation), headache and allergic reactions. Also, the children were visited every month by a pediatrician.

###  Randomization

Convenience sampling was done for all participants who referred to out-patient child and adolescent clinic of *Khorshid* Hospital of *Isfahan*, Iran. Sixty four eligible patients were randomized in two parallel groups. A non-stratified block randomization list (equal blocks) was created by an epidemiologist using Number Cruncher Statistical System. Then, the eligible children were assigned into two arms by the researcher according to the randomized list.

###  Ethical Issues

 This clinical trial is in compliance with the Declaration of Helsinki (1989 revision). It was reviewed, accepted, and monitored by the Ethics Committee of Yazd Shahid Sadoughi University of Medical Sciences (identification number: 17/1/296633/p). Moreover, the trial was registered by Iranian Registry of Clinical Trials with the following code: IRCT20180303038930N1. Informed consent form was signed by the parents to enroll their child in the study. In addition, verbal consent was obtained from the participating children.

###  Statistical Methods

 Data analysis was done using Statistical Package for the Social Sciences software, version 15 (SPSS Inc., Chicago, IL, USA). The mean and standard deviation of the dependent variables were reported separately for each group. The Mann–Whitney U test was used for statistical comparison of data before and after the intervention. The Wilcoxon test was used to find out the changes in the outcomes between the two groups of the trial. P-values more than 0.05 were considered insignificant.

## Results

###  Patients’ Enrollment

**Figure 1:**
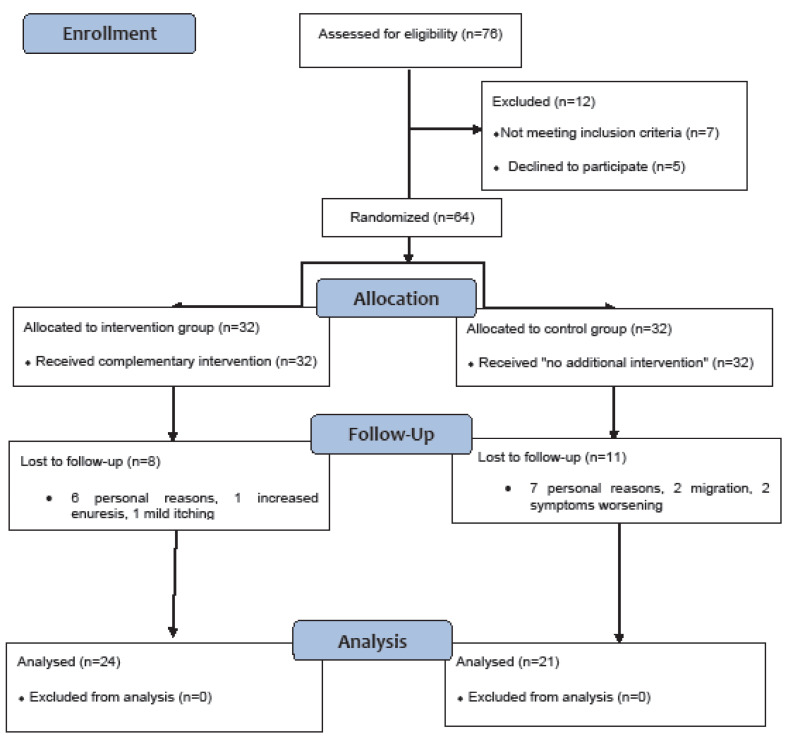
Flow diagram of the groups' allocation, intervention, follow-up, and the analysis in both groups of the study

 From October 2016 to May 2017, seventy six children and adolescents were assessed for eligibility. Sixty-four eligible participants were randomized into two groups (32 members), (allocation ratio 1:1). During the study, 8 patients in the intervention group and 11 in the control group were excluded due to lack of willingness to continue cooperation. Figure-1 is the CONSORT flowchart of the study groups’ allocation, intervention, follow up, and analysis.

###  Baseline Characteristics

 The mean age of the children in the trial was 9.39 (±1.99) and 9.37(±2.37) years in the intervention and control groups, respectively. No significant difference was shown between the two groups of the study (P=0.922). Among the children, 19 boys and 2 girls were in the control group and 18 boys and 6 girls were in the intervention group. No significant differences were observed in sex distribution among the two groups of the trial (P=0.168). All variables were compared with baseline scores and there was no significant difference between the study groups at baseline (P>0.05, Tables-1 and 2).

###  Clinical Response

 As shown in Table-1, parent reported hyperactivity scale showed a significant decrease in the intervention group from 7.19(±2.20) to 6.00(±2.00) compared to the control group, from 7.61(±1.75) to 8.00(±1.68) (P=0.044). However, no significant differences were observed in other scales of parent-reported SDQ between the two groups. Teacher-reported SDQ scores are presented in Table-2. There was only a slight worsening of prosocial behavior score in the control group when compared to its baseline score. Nevertheless, no significant between group differences were observed in the scales of teacher-reported SDQ. According to the results of CPT, there was a significant improvement in the intervention group regarding attention and focus score, from 8.83(±4.22) to 11.12(±4.37) (P=0.013, Table-3).

###  Safety and Tolerability

No significant side effects were observed with whey protein. In one case, *ma’aljobon* exacerbated the itching of the neck, and, in another case, whey protein exacerbated enuresis.

## Discussion

In this open-label randomized double arm controlled clinical trial, the effect of *ma’aljobon* in management of ADHD was investigated and the results showed that the children’ hyperactivity (according to the parent’s report) was significantly improved after the study in the intervention group in comparison to the control group. Also, whey consumption resulted in a significant improvement in the attention and focus score after 2 months. To the best of our knowledge, this is the first study that examined the efficacy of *ma’aljobon* as a kind of whey protein in management of children with ADHD. In recent decades, many studies have been done on the biological and therapeutic effects of whey protein. This diary product has been studied in different clinical trials on a wide variety of diseases such as cancer, AIDS, hepatitis, cardiovascular diseases, and osteoporosis [17,28-34]. Conversion of intracellular amino acid cysteine into glutathione, as an intracellular antioxidant, is described as the primary mechanism of whey protein. Whey is also rich in amino acids containing sulfosalemic: cysteine and methionine. Whey protein increases the cellular activity by increasing the concentration of amino acids inside the cell and converting them into glutathione [35,36]. According to previous studies, cognitive activity of the brain increases when the serotonergic activity of the brain increases. Serotonin precursor tryptophan uptake into the brain depends on the nutrients that influence the availability of tryptophan. Moreover, a diet-induced increase in tryptophan possibly raises the brain serotonergic activity and recovers the cognitive performance, mainly in high stress-vulnerable subjects. Whey protein is rich in alpha-lactalbumin. Therefore, it can increase the ratio of plasma tryptophan to the sum of the other large neutral amino acids and improve cognitive performance. Markus *et al*. demonstrated that whey protein could improve cognitive and coping ability in severe stress cases [23].

###  Study Limitations

 For a more comprehensive understanding of the results, we should consider the limitations of our study. The small number of participants was the main limitation of this study. Considering the trend of the changes in the outcome measures of the study, if we had a larger sample size, we would possibly reach more reliable results. Lack of a placebo group was another limitation of this study which caused it to be designed as an open-label model and its own biases. In addition, it would have been beneficial for increasing the reliability of the results to add some other questionnaires to assess both symptoms and functional impairment of children with ADHD. This should be added as a suggestion for future research. Using only a fixed dose of whey protein and the short period of follow-up were the other limitations. Also, recording daily diet, parents’ level of education, and the socioeconomic status of the families and their number of children should be kept in mind for future research. They may affect the intervention’s outcome as possible confounding factors. Although two months is the standard time for evaluating ADHD treatment, longer follow-up of patients may have different results about the effectiveness of whey protein in the treatment of ADHD. Due to the chronic nature of the ADHD and the need for long-term treatment for patients, a long time intervention is required.

## Conclusion

Results of this trial showed that *ma’aljobon* might be considered as a complementary therapy for improving hyperactivity, attention and focus of children with ADHD. However, it seems that it has no effect on the emotional symptoms, conduct problems, peer problems, and prosocial behavior. Hence, conducting studies in a longer period of time with larger trial sample size and removing our limitations are required for making more consistent opinions on whey protein use in ADHD.

## Acknowledgement

 This study was a part of a PhD thesis by Dr. Zeinab Mostajeran. The authors thank the research vice chancellery of Yazd University of Medical Sciences for funding supports. The authors also thank Dr N. Shokrpour for the linguistic editing of this manuscript.

## Conflict of Interest

 None declared.

**Table 1:** Mean Scores of Parent Reported Strengths and Difficulties Questionnaire of the Two Groups of the Study before and after the Intervention

**Strengths and Difficulties Questionnaire (SDQ) Scales**	**Study Groups**	**Before (Mean ± SD)**	**After** **(Mean ± SD)**	**P-value** ^*^	**P-value** ^**^
**Emotional Symptoms Scale**	Intervention	5.142±2.22	4.71±2.42	0.49	0.88
	control	4.50±1.94	4.21±2.25	0.57	
	P-value***	0.34	0.50		
**Conduct Problem Scale**	Intervention	4.09±2.27	4.95±2.60	0.42	0.55
	control	4.44±2.20	4.38±2.25	0.67	
	P-value***	0.63	0.48		
**Hyperactivity Scale**	Intervention	7.19±2.20	6.00±2.00	0.09	0.04
	control	7.61±1.57	8.00±1.68	0.77	
	P-value***	0.51	0.002		
**Peer Problem Scale**	Intervention	4.38±1.53	3.73±1.82	0.50	0.66
	control	3.88±1.90	4.16±2.40	0.95	
	P-value***	0.39	0.54		
**Prosocial behavior Scale**	Intervention	7.33±1.87	7.47±1.77	0.43	0.62
	control	7.64±2.59	6.88±2.57	0.09	
	P-value***	0.68	0.42		

^*^ Wilcoxon test ^**^ Mann-Whitneytest ***Independent t-test

**Table 2:** Mean Scores of Teacher Reported Strengths and Difficulties Questionnaire of Two Groups of the Study before and after the Intervention

**Strengths and Difficulties Questionnaire( SDQ) Scales**	**Study Groups**	**Before (Mean ± SD)**	**After** **(Mean ± SD)**	**P-value** ^*^	**P-value** ^**^
**Emotional Symptoms Scale**	Intervention	3.95±2.92	4.00±2.11	0.89	0.43
			
control	3.07±2.09	2.85±2.14	0.52
	P-value***	0.31	0.12		
**Conduct Problem Scale**	Intervention	2.75±2.33	3.40±2.13	0.15	0.06
	control	4.00±2.42	3.57±2.37	0.36	
	P-value***	0.13	0.83		
**Hyperactivity Scale**	Intervention	6.31±2.49	6.22±2.36	0.90	0.18
	control	7.20±2.42	6.00±2.67	0.05	
	P-value***	0.29	0.78		
**Peer Problem Scale**	Intervention	4.20±1.90	3.85±2.35	0.67	0.55
	control	4.00±2.36	4.33±2.73	0.59	
	P-value***	0.78	0.58		
**Prosocial behavior Scale**	Intervention	5.77±2.97	6.71±2.59	0.32	0.60
	control	5.42±2.62	6.64±2.67	0.01	
	P-value***	0.73	0.93		

^*^ Wilcoxon test ^**^ Mann-Whitneytest ***Independent t-test

**Table 3:** Mean Scores of Conners’ Continuous Performance Test (CPT) of the Two Groups of the Study before and after the Intervention

**Conners’ Continuous Performance Test (CPT) scores**	**Study Groups**	**Before** **(Mean ±SD)**	**After** **(Mean ±SD)**	**P-value** ^*^	**P-value** ^**^
Attention and focus	Intervention	8.83±4.22	11.12±4.37	0.013	0.33
	control	8.60±5.08	9.95±4.80	0.392	
	P-value***	0.86	0.40		
Impulsivity	Intervention	22.79±26.56	21.83±24.09	0.414	0.12
	control	13.10±20.64	13.70±21.40	0.137	
	P-value***	0.19	0.24		
Vigilance	Intervention	1.01±0.30	0.99±0.28	0.648	0.11
	control	0.90±0.42	1.18±0.39	0.218	
	P-value***	0.36	0.07		

^*^ Wilcoxon test ^**^ Mann-Whitneytest ***Independent t-test
